# New York Pathological Society

**Published:** 1856-02

**Authors:** 


*New York Pathological Society.*—Dr. Markoe exhibited a specimen of *fracture of the cervix femoris*, taken from a girl nine years old, brought into the New York Hospital, with injuries received in a fall from a sixth-story window. Attention was especially directed to the left hip. The limb was shortened half an inch—knee slightly flexed, and the toe inclined to be everted. On rotation, the trochanter moved on a very short radius; and on drawing down the limb, and making rotation, crepitus was abundant and distinct. The point of the trochanter followed the movements of the shaft, showing it not to be detached. Taken in connection with the manner of the accident, and the age of the patient, these symptoms seemed to indicate an *extra capsular fracture* of the neck of the *femur*. It became a matter of some interest to ascertain, whether the case would throw any light upon the *pathological law*, most clearly elucidated by Robert William Smith, of Dublin, that in *extra capsular fracture* of the *cervix fermoris*, there is always a wedge action of the upper upon the lower fragment, whereby the trochanter is split more or less completely into several fragments. She died the next day, from the injuries sustained.

*Postmortem examination.* On inspection, a fracture is observed just external to the capsule, separating the neck from the *femur*. The specimen also shows clearly another fracture, separating the point of the trochanter from the shaft; a separation, which, when we look sidewise at the specimen, is evidently produced by the wedge action alluded to; this, however, was not at first so readily seen, because the aponeurotic covering of the bone was not entirely lacerated, thus keeping the fragment in connection with the shaft, and preventing this second fracture from being discovered before death. It is particularly interesting to observe that the separation has taken place at the line of junction of the epiphysis, the upper fragment being in fact the epiphysis, only partly ossified. It is the youngest case of fracture of the *cervix femoris* that he has ever observed.—*New York Med. Times.*

